# A new species of *Mastigodiaptomus* Light, 1939 from Mexico, with notes of species diversity of the genus (Copepoda, Calanoida, Diaptomidae)

**DOI:** 10.3897/zookeys.637.10276

**Published:** 2016-11-30

**Authors:** Martha Angélica Gutiérrez-Aguirre, Adrián Cervantes-Martínez

**Affiliations:** 1 Universidad de Quintana Roo (UQROO), Unidad Cozumel, Av. Andrés Quintana Roo s/n, 77600, Cozumel, Quintana Roo México

**Keywords:** COI gene, freshwater, Mexico, morphology, taxonomy

## Abstract

A new species of the genus *Mastigodiaptomus* Light, 1939, named *Mastigodiaptomus cuneatus* **sp. n.** was found in a freshwater system in the City of Mazatlán, in the northern region of Mexico. Morphologically, the females of this new species are distinguishable from those of its congeners by the following combination of features: the right distal corner of the genital double-somite and second urosomite have a wedge-shaped projection, the fourth urosomite has no dorsal projection and its integument is smooth. The males are distinct by the following features: the right caudal ramus has a wedge-shaped structure at the disto-ventral inner corner; the basis of the right fifth leg has one triangular and one rounded projection at the distal and proximal margins, respectively, plus one hyaline membrane on the caudal surface close to the inner margin; the aculeus length is almost the width of the right second exopod (Exp2); and the frontal and caudal surfaces of the right Exp2 are smooth. Furthermore, the analysis of the COI gene of *Mastigodiaptomus cuneatus* sp. n. has revealed that *Mastigodiaptomus albuquerquensis* (Herrick, 1895) is its nearest congener, with 18.64% of genetic distance. A key for the identification of the known species of the genus is provided.

## Introduction

Diaptomidae G. O. Sars, 1903 is one of the most common families of freshwater copepods worldwide, some genera of this family have restricted distributional patterns and present endemic forms, as the genus *Mastigodiaptomus* Light, 1939. Recent studies of this genus in the Neotropical region have added new species of these diaptomids (Gutiérrez-Aguirre and Cervantes-Martínez 2013) or the morphological and genetic analysis have produced recognition of species such as *Mastigodiaptomus patzcuarensis* (Kiefer, 1938) (Gutiérrez-Aguirre et al. 2014). Currently ten species of *Mastigodiaptomus* are known: *Mastigodiaptomus purpureus* (Marsh, 1907), *Mastigodiaptomus texensis* (M. S. Wilson, 1953), *Mastigodiaptomus amatitlanensis* (M. S. Wilson, 1941), *Mastigodiaptomus albuquerquensis* (Herrick, 1895), *Mastigodiaptomus patzcuarensis* (Kiefer, 1938), *Mastigodiaptomus montezumae* (Brehm, 1955), *Mastigodiaptomus nesus* Bowman, 1986, *Mastigodiaptomus maya* Suárez-Morales & Elías-Gutiérrez, 2000, *Mastigodiaptomus reidae* Suárez-Morales & Elías-Gutiérrez, 2000, and *Mastigodiaptomus suarezmoralesi* Gutiérrez-Aguirre & Cervantes-Martínez, 2013.

The fine structural features of the anatomy of the females and males of freshwater copepods are informative for species recognition. Researchers that have developed this idea concerning free-living copepods are Van de Velde (1984) and Hołyńska (2000) in the Cyclopidae genus *Mesocyclops*; and Alekseev et al. (2006) in the Cyclopidae genus *Eucyclops*, particularly the *Eucyclops serrulatus* species complex. Bowman (1986) recognized morphologically similar species within the *Mastigodiaptomus* genus using differences in the integument of prosomal wings and fifth legs of both sexes. Empirical evidence gathered from several species of freshwater crustaceans has shown that these morphological differences are consistent with reproductive isolation (Alekseev et al. 2006), genetic differentiation (Quiroz-Vázquez and Elías-Gutiérrez 2009; Gutiérrez-Aguirre et al. 2014), or both when they are probed (Montiel-Martínez et al. 2008).

In the present work is provided an illustrated description of both sexes of one new species of the genus *Mastigodiaptomus*; it was found in the northern region of Mexico in a field collection carried out in 2014. The analysis was based upon the detailed micro-structure of the antennules, fifth legs, and prosomal integument. In addition, the sequences of the mitochondrial COI gene were used to assess the genetic divergence between the new species and seven congeners to incorporate molecular information into the species description.

## Methods

Morphological analysis. The cephalic appendages, swimming legs and urosome of *Mastigodiaptomus cuneatus* sp. n. were examined using light microscopy and illustrated with the aid of a camera lucida. The specimens were dissected and appendages were mounted in glycerine.

The terminology and abbreviations used for the armament of each appendage and structure are based on Huys and Boxshall (1991):

**s** setae

**sp** spine

**sps** spiniform process

**ae** aesthetasc

**Enp1–Enp^n^** first to “n” endopodal segment

**Exp1–Exp^n^** first to “n” exopodal segment

**P1–P5** Legs 1–5

The type material was deposited at the Colección de Referencia de El Colegio de la Frontera Sur (ECOCH-CHZ) Chetumal, México and the Colección Nacional de Crustáceos (CNCR) del Instituto de Biología, Universidad Nacional Autónoma de México.

Molecular analysis. Specimens (2 females, 3 males, and 3 copepodites) were preserved after capture in 96% ethanol and prepared for barcoding following standard methods. DNA was extracted using the HOTSHOT method (Montero-Pau et al. 2008). A segment of the COI gene was amplified using the LCOI490 and HCO2198 primers or the Zplank primers, as suggested by Folmer et al. (1994) and Prosser et al. (2013), respectively. The preparation of the PCR 12.5 µL PCR reaction mixture and visualization of PCR products was performed as described by Gutiérrez-Aguirre et al. (2014) and Montiel-Martínez et al. (2008). Sequence analysis was carried out at the Chetumal Node of the MEXBOL (El Colegio de la Frontera Sur).

Additionally, a search of GenBank and the public data of the Barcode of Life Data System (BOLD) produced sequences of *Mastigodiaptomus albuquerquensis*, *Mastigodiaptomus patzcuarensis*, Mastigodiaptomus cf. albuquerquensis (accession numbers for BOLD and GenBank in Gutiérrez-Aguirre et al. 2014), *Mastigodiaptomus texensis*, Mastigodiaptomus cf. nesus, *Mastigodiaptomus montezumae*, Mastigodiaptomus cf. reidae, and *Mastigodiaptomus reidae* (accession numbers for BOLD and sampled site are shown in Table 1).

**Table 1.** Localities and sequence access for specimens surveyed herein, recorded in Mexico and Canada.

Species	Sample ID BOLD	Locality	Lat N	Long W
*Hesperodiaptomus arcticus* (Marsh, 1920)	CHU-CRU-0777	Churchill, Manitoba, Canada	58.772	93.844
*Hesperodiaptomus arcticus* (Marsh, 1920)	BIOUG01701-E09	Churchill, Manitoba, Canada	58.7715	93.844
Mastigodiaptomus cf. nesus	Cala039	Minicenote, Quintana Roo, Mexico	18.647	88.412
Mastigodiaptomus cf. nesus	Cala041	Minicenote, Quintana Roo, Mexico	18.647	88.412
Mastigodiaptomus cf. nesus	ZMXII-508	La Esperanza, Q. Roo, Mexico	19.471	88.029
Mastigodiaptomus cf. reidae	ZPLMX581	Kohunlich, Q. Roo, Mexico	18.447	88.825
*Mastigodiaptomus cuneatus* sp. n.	MAGA-0156	El Camarón, Sinaloa, Mexico	23.236	106.438
*Mastigodiaptomus montezumae* (Brehm, 1955)	HE-150a	Timilpan, Edo. Mex., Mexico	19.887	99.739
*Mastigodiaptomus montezumae* (Brehm, 1955)	HE-151a	Timilpan, Edo. Mex., Mexico	19.887	99.739
*Mastigodiaptomus montezumae* (Brehm, 1955)	HE-154a	Timilpan, Edo. Mex., Mexico	19.887	99.739
*Mastigodiaptomus montezumae* (Brehm, 1955)	ZPLMX210	km 25, Edo. Mex., Mexico	19.486	99.745
*Mastigodiaptomus reidae* Suárez-Morales & Elías-Gutiérrez, 2000	ZPLMX224	Kohunlich, Q. Roo, Mexico	18.447	88.825
*Mastigodiaptomus reidae* Suárez-Morales & Elías-Gutiérrez, 2000	ZPLMX579	Kohunlich, Q. Roo, Mexico	18.447	88.825
*Mastigodiaptomus reidae* Suárez-Morales & Elías-Gutiérrez, 2000	ZPLMX578	Kohunlich, Q. Roo, Mexico	18.447	88.825
*Mastigodiaptomus texensis* (M. S. Wilson, 1953)	Cala012	Boca del Puma, Q. Roo, Mexico	20.871	87.055
*Mastigodiaptomus texensis* (M. S. Wilson, 1953)	Cala016	Boca del Puma, Q. Roo, Mexico	20.871	87.055
*Mastigodiaptomus texensis* (M. S. Wilson, 1953)	Cala065	Verde Lucero, Q. Roo, Mexico	20.866	87.077
*Mastigodiaptomus texensis* (M. S. Wilson, 1953)	Cala066	Verde Lucero, Q. Roo, Mexico	20.866	87.077
*Mastigodiaptomus texensis* (M. S. Wilson, 1953)	Cala069	Verde Lucero, Q. Roo, Mexico	20.866	87.077

These sequences were downloaded from the BOLD project files *Mastigodiaptomus* of Mexico (MALB), Microcrustacean from Mexico (MCM), and Zooplankton II (ZPII) and compared with our sequence of *Mastigodiaptomus cuneatus* sp. n., this latter sequence is into the project MCM. In these project files, the electropherograms, sequence data, photographs, primers data, and collection details are available (on the Barcode of Life Data System http://www.boldsystems.org). Fifty-two COI gene sequences > 500 bp were used for the analysis, and BOLD Aligner and the ID Tree using the model Kimura 2 parameter (K2P; Kimura 1980) were utilized to obtain the ID Tree. Two sequences of *Hesperodiaptomus arcticus* (Marsh, 1920) (Calanoida: Diaptomidae) were used as an outgroup.

## Results

### Descriptive section Order Calanoida G. O. Sars, 1903 Family Diaptomidae G. O. Sars, 1903 Subfamily Diaptominae Kiefer, 1932 Genus *Mastigodiaptomus* Light, 1939

#### Mastigodiaptomus cuneatus sp. n.

Taxon classificationAnimaliaCalanoidaDiaptomidae

http://zoobank.org/FADC3B97-FB6F-4559-B71B-EB6F76A3246F

**Figure 1.**
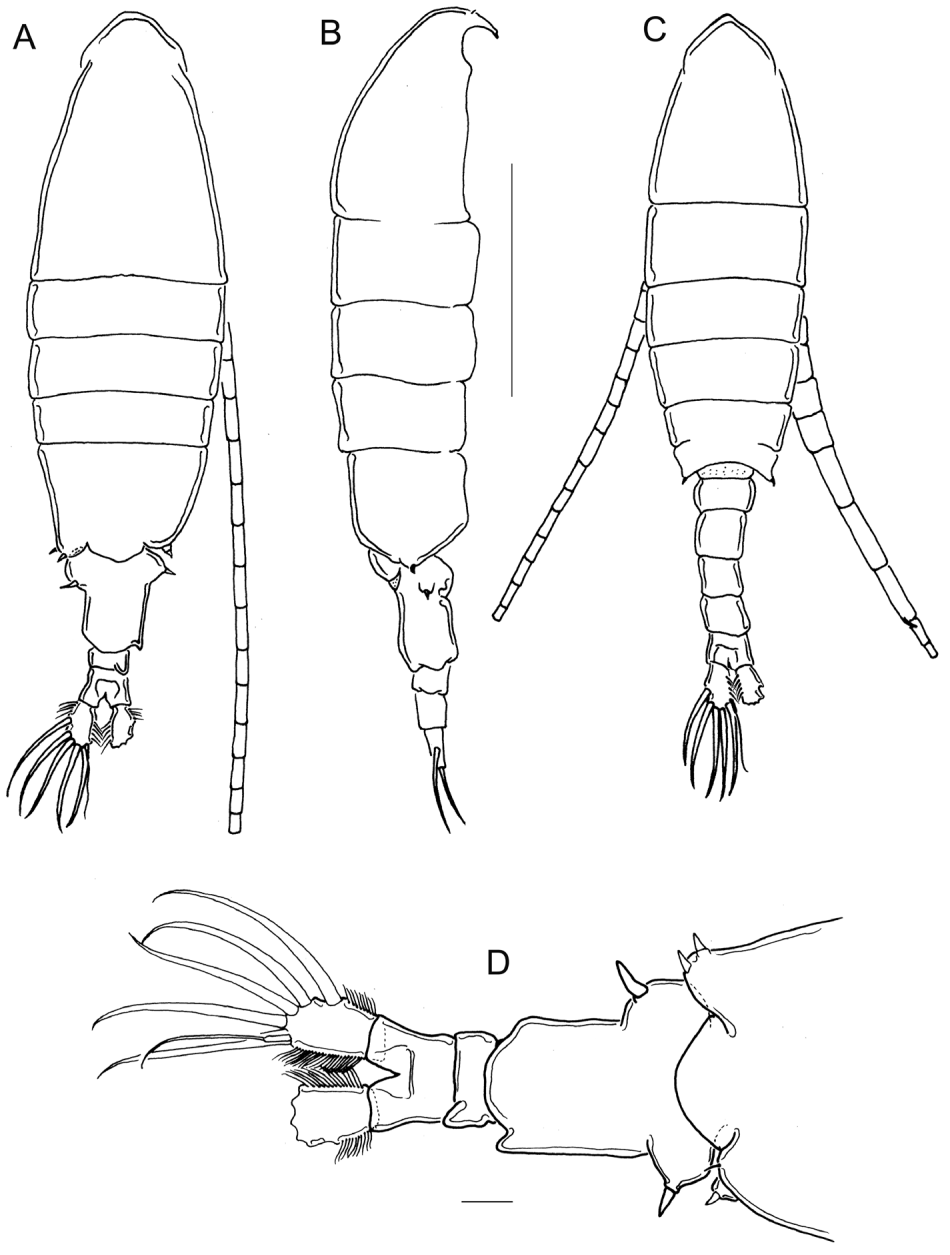
*Mastigodiaptomus cuneatus* sp. n. Adult female, holotype (**A, B, D**) and adult male, allotype (**C**) **A** Habitus, dorsal **B** Habitus, lateral **C** Habitus, dorsal **D** Fifth pediger and urosome, dorsal. Scale bars: 500 µm (**A–C**); 50 µm (**D**).

**Figure 2.**
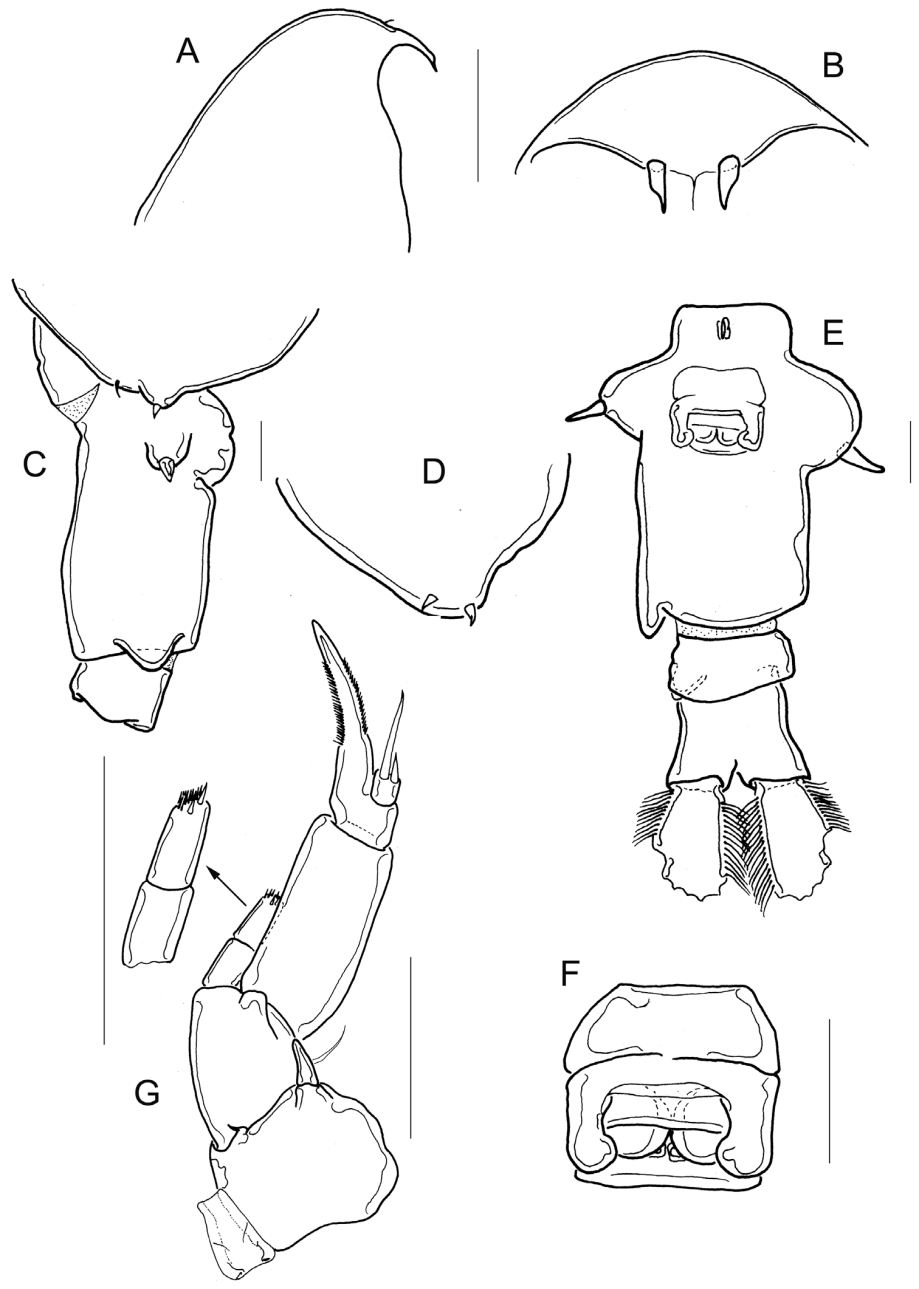
*Mastigodiaptomus cuneatus* sp. n. Adult female, holotype. **A** Rostrum, lateral **B** Rostrum, frontal **C** Wing of fifth pediger, genital double-somite, and second urosomite, lateral right view **D** Wing of fifth pediger, lateral, left view **E** Urosome, ventral **F** Genital field **G** Fifth leg, frontal. Scale bars: 50 µm.

**Figure 3.**
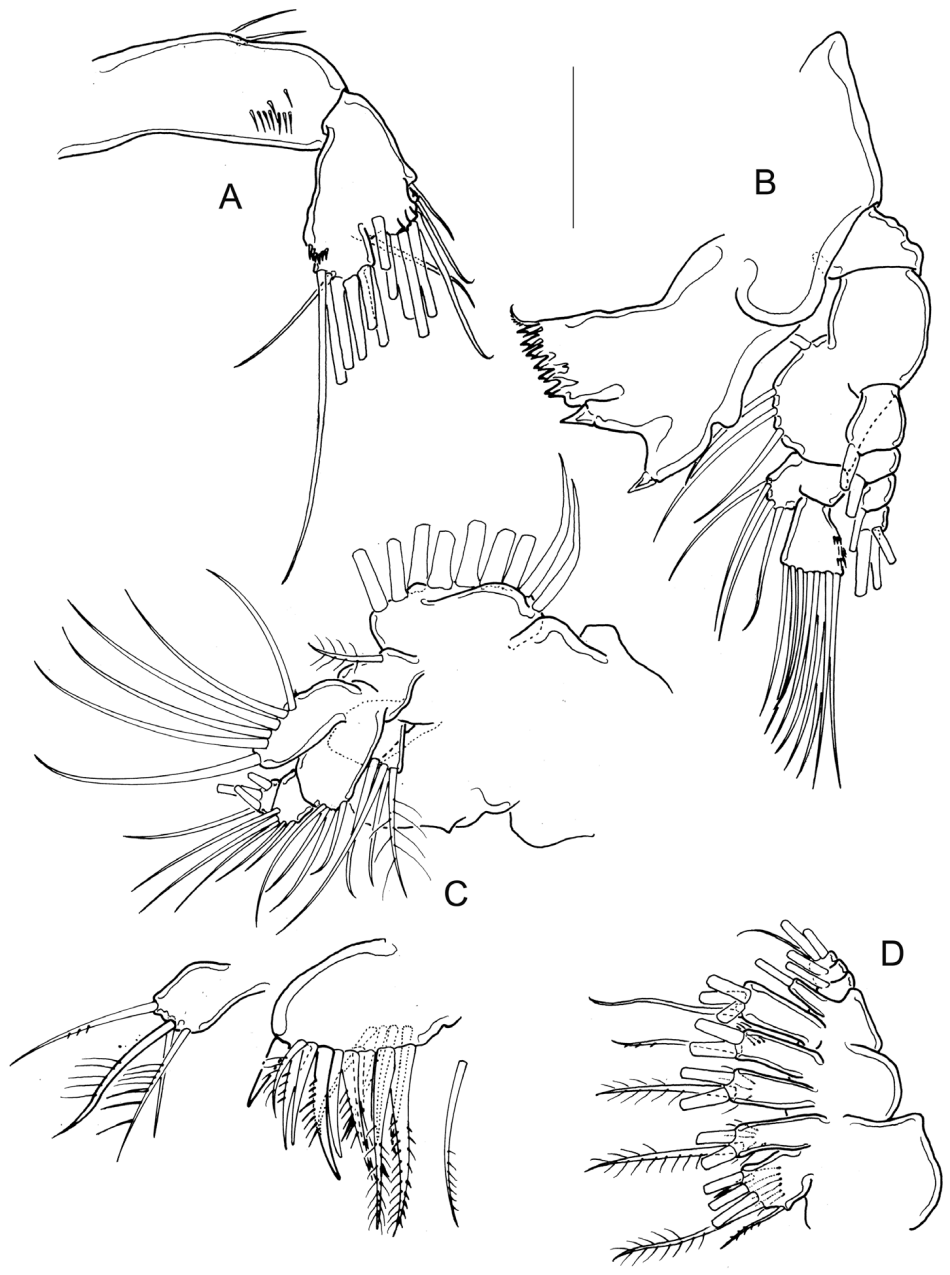
*Mastigodiaptomus cuneatus* sp. n. Adult female, holotype. **A** Enp1 and Enp2 of antenna **B** Mandible **C** Maxillule, coxal endite and praecoxal arthrite separated **D** Maxilla. Scale bar: 50 µm.

**Figure 4.**
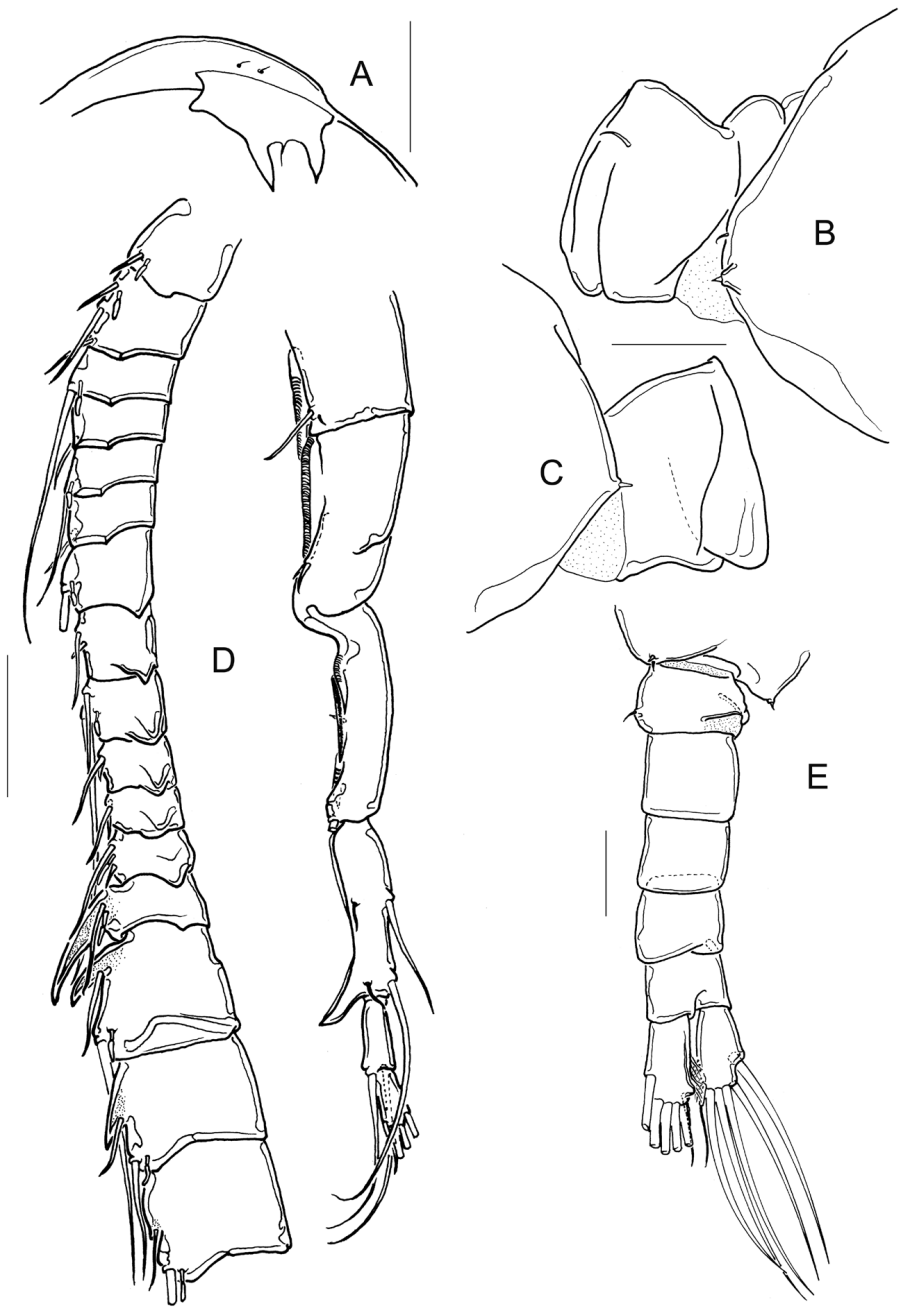
*Mastigodiaptomus cuneatus* sp. n. Adult male, allotype. **A** Rostrum, frontal **B** Wing of fifth pediger and first urosomite, lateral right view **C** Wing of fifth pediger and first urosomite, lateral left view **D** Right antennule **E** Fifth pediger and urosome, ventral. Scale bars: 50 µm (**A–C**); 100 µm (**D, E**).

**Figure 5.**
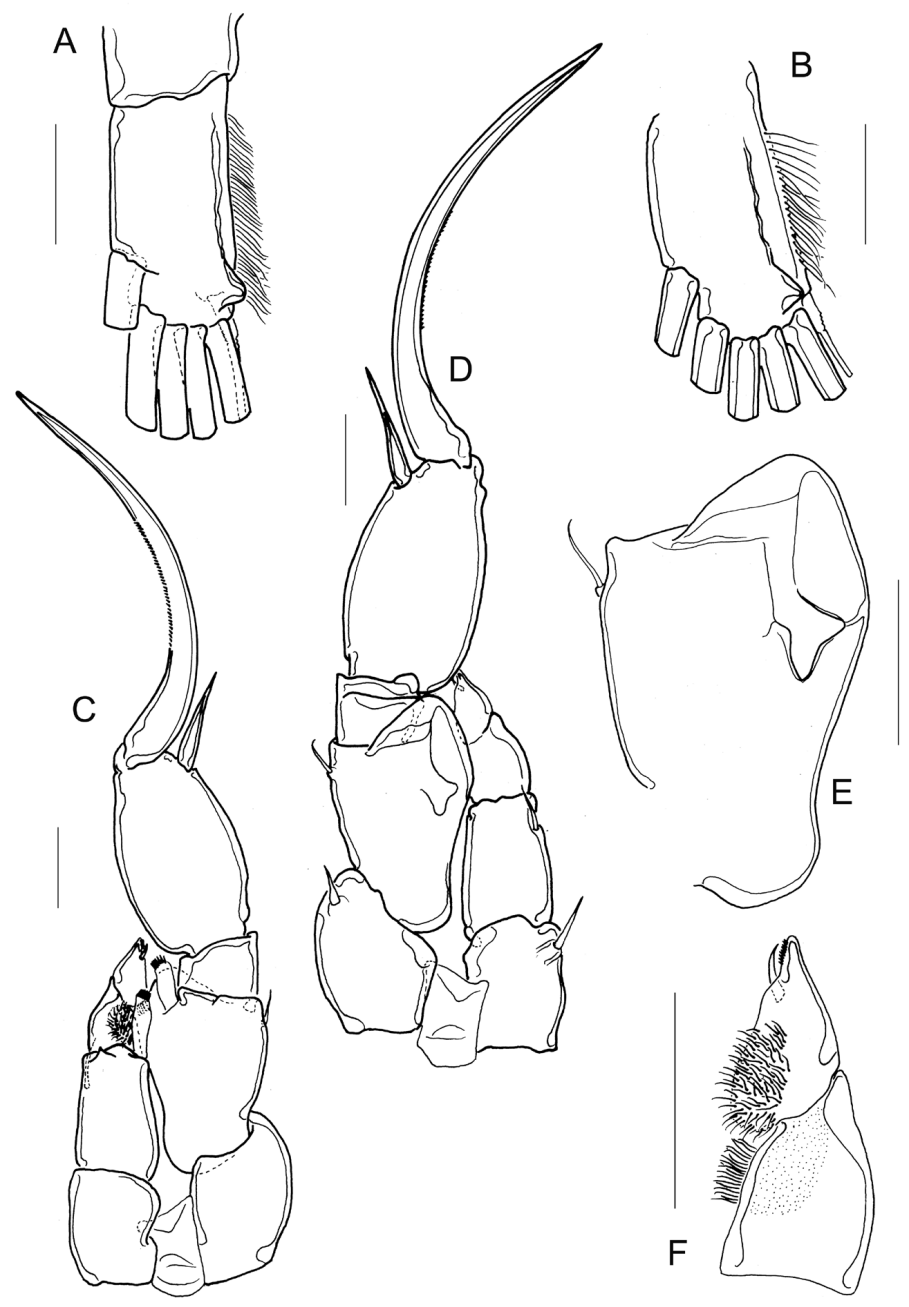
*Mastigodiaptomus cuneatus* sp. n. Adult male, allotype. **A** Right furcal ramus, semi-lateral **B** Right furcal ramus, ventral **C** Fifth leg, frontal **D** Fifth leg, caudal **E** Fifth leg, right basis, caudal **F** Fifth leg, left Exp1 and Exp2. Scale bars: 50 µm.

Figures 1 , 2 , 3 , 4 , 5

##### Holotype.

One adult female dissected on one slide: ECOCH-Z-09339. Collected 28.VIII.2014. Collectors: A. Cervantes-Martinez, N. Hernández López, M. Bastidas, and J. Aguilar Rubio.

##### Allotype.

One adult male dissected on one slide: ECOCH-Z-09340. Collected 28.VIII.2014. Same collectors.

##### Paratypes.

Four adult females and five adult males preserved in 90% ethanol with a drop of glycerine. ECOCH-Z-09341. Collected 28.VIII.2014. Same collectors.

Two adult females and two adult males preserved in 90% ethanol: CNCR-31861. Collected 28.VIII.2014. Same collectors.

##### Type locality.

A lagoon called Laguna El Camarón in Avenida Insurgentes, Mazatlán, Sinaloa City, México; 23°14'10"N; 106°26'18"W.

##### Etymology.

The name of the species means “wedged” in Latin and refers to the chitinous protuberance present on the right disto-lateral corner of the first and second urosomites in females, and on the right caudal ramus on the ventral surface in males.

##### Diagnosis.

Adult female: Cuticle surfaces of prosomal somites smooth dorsal and laterally (Fig. 1A, B). Antennules tip reaching beyond the caudal rami. Right wing of fifth pediger with one tiny dorsal spinule plus one stout ventral spine; left wing with two spines (Fig. 1A, D). No dorsal process on the last thoracic somite (Fig. 1B). Genital double-somite and second urosomite with one lateral wedge each on distal margin on the right side (Fig. 1D). Genital double-somite asymmetric and laterally bulbose; each bulb bearing a stout lateral spine (Fig. 1D). Short spines on the rostrum, which are less than 3 times longer than wide (Fig. 2A, B). Endopodite of fifth leg 2-segmented with a row of short spinules (arranged in one oblique line) flanked by 2 larger spinules; Exp3 of the fifth leg bearing 2 apical spines (Fig. 2G).

Adult male: The cuticle surfaces of prosomal somites are smooth dorsally and laterally (Fig. 1C). Right antennule 22-segmented, with one fang-like process on antepenultimate segment, which is less than half the length of the penultimate segment (Fig. 4D). Right antennule with spiniform process on segments 10, 11, and 13 to 16 (Fig. 4D). Inner margin of caudal ramus fringed by long hair-like setae (Fig. 1C). One wedge on distal margin of right caudal ramus on the ventral surface (Fig. 4E). Left and right coxae of the fifth leg have long, acute spines on lateral margins; apical spine of right Exp2 with tiny spinules along medial margin (Fig. 5D). One triangular and one rounded projection located at distal and proximal margins of the right basis, respectively, plus one hyaline membrane on caudal side (Fig. 5D). The aculeus length is almost the width of right Exp2. Left and right endopods one-segmented, with apical spinules (Fig. 5C).

##### Description adult female.

Smooth prosomal somites; body length 1500-1700 µm in paratypes including caudal ramus, *n* = 6 (Fig. 1A, B). Spines on rostrum 2.8-3.0 times longer than wide (Fig. 2B).

Antennule: 25-segmented, extending beyond the caudal ramus (Fig. 1A). Armature details with setae, spines, or aesthetasc in the next order: (1)1s + 1ae; (2)3s+1ae; (3)1s+1ae; (4)1s; (5)1s + 1 ae; (6)1s; (7)1s+1ae; (8)1s +1 sp; (9)2s + 1ae; (10)1s; (11)1s+1ae; (12)1s + 1ae + 1sp; (13)1s; (14)1s+1ae; (15)1s; (16)1s+1ae; (17)1s; (18)1s; (19)1s+1ae; (20)1s; (21)1s; (22)1s + 1ae; (23)1s + 1ae; (24)2s; (25)4s + 1ae.

Antenna: Coxa with one long seta; basis with 2 setae; Exp 7-segmented with 1, 3, 1, 1, 1, 1, 4 setae, respectively. Enp 2-segmented, Enp1 with 2 setae plus a row of spine-like setae. Inner lobe of Enp2 bearing 9 long setae, outer lobe with 7 setae and a group of tiny spinules (Fig. 3A).

Mandible: Eight teeth on gnathobase (6 of these teeth bifid) with a movable seta at tip. Rectangular, nude coxa. Basis with 4 long setae. Enp two-segmented, Enp1 with 4 setae, Enp2 with 2 distal pectens and 9 long setae. Exp 4-segmented, with 1, 1, 1, and 3 long setae, respectively (Fig. 3B).

Maxillule (Fig. 3C): Coxal epipodite elliptical, bearing 9 long setae. Short basal exite, one-setulated; Exp plate-like with 6 long setae. Enlarged basal endite with 4 setae and 2-segmented endopodite: Enp1 and Enp2 with 5 and 4 setae, respectively. Basis rectangular with 4 setae; coxal endite quadrangular 4-setulated. Praecoxal arthrite with 15 spiniform setae, 11 anterior, 4 posterior.

Maxilla (Fig. 3D): First praecoxal lobe with 4 long setae and 1 lateral short seta; plus long setules and 1 spinule on posterior side. Second praecoxal lobe 3-setulated. Two coxal lobes with 3 long setae each. All the praecoxal and coxal lobes with long setules located posteriorly. First basal lobe with 4 setae, second basal lobe with 1 seta. Three-segmented Enp, with 1, 1, and 3 setae, respectively.

Maxilliped (not figured): Same structure as described and illustrated for *Mastigodiaptomus albuquerquensis* and *Mastigodiaptomus patzcuarensis* (see Gutiérrez-Aguirre et al. 2014).

P1-P4: The number of segments on endopods and exopods of P1 to P4, as described for copepods that belong to the Diaptomidae family (Dussart and Defaye 1995). Armature formula of swimming legs as shown in Table 2 including Schmeil’s organ on Enp2P2.

**Table 2.** Setation formula of the swimming legs in *Mastigodiaptomus cuneatus* sp. n. (spine in Roman numerals, seta in Arabic numerals).

	Coxa	Basis	Exp	Enp
P1	0-1	0-0	I-1; 0-1; I-3-2	0-1; 1-2-3
P2	0-1	0-0	I-1; I-1; I-3-3	0-1; 0-2; 2-2-3
P3	0-1	0-0	I-1; I-1; I-3-3	0-1; 0-2; 2-2-3
P4	0-1	1-0	I-1; I-1; I-3-3	0-1; 0-2; 2-2-3

Fifth pediger wings and urosome: Right wing of fifth pediger with 2 spines, one dorsal and one ventral (Fig. 2C), left wing with 2 equal spines (Fig. 2D). Genital double-somite and second urosomite each with projections at right distal corner (Fig. 2C); these projections wedge-like in dorsal (Fig. 1D) and ventral views (Fig. 2E). Genital double-somite 1.2 ± 0.1 times longer than wide, lateral margins bulbous and with strong spines; right spine inserted laterally, more proximal than left spine (Fig. 2E). Caudal ramus with long hair-like setules on medial and lateral margins (Fig. 2E). Genital field quadrangular with parallel lateral margins (Fig. 2F).

Fifth leg (Fig. 2G): Coxa with a large spine on distal margin, basis quadrangular with a blunt projection on distal margin and one slender lateral seta. Exp1 1.66 times longer than 2-segmented endopod. Second endopodal segment with a row of spinules at the tip arranged in an oblique line, flanked by 2 larger spinules. Exp2 separated, with one long and one short spine. Exp3 with spinules on distal medial and lateral margins.

##### Description adult male.

Prosomites smooth in dorsal view; left antennule reaching anal somite. Body length 1400-1500 µm in paratypes including caudal ramus, *n* = 7 (Fig. 1C). Short spines on rostrum 2.7-2.8 times longer than wide (Fig. 4A).

Right antennule (Fig. 4D): 22-segmented, each segment armed with setae, spines, spiniform process, or aesthetasc in the following order: (1)1s+1ae; (2)2s+2ae; (3)1s+1ae; (4)1s; (5)1s+1ae; (6)1s; (7)1s+1ae; (8)1s+1sp; (9)2s+1ae; (10)1s+1sps, (11)1s+1sps; (12)1s+1ae+1sp; (13)1s+1ae+1sps; (14)2s +1ae+1sps; (15)2s +1ae+1sps; (16)2s+ 1ae+1sps; (17)1s+1sp; (18)1s+1sp; (19)1s+1ae; (20)4s; (21)2s; (22)3s+1ae. Segments 17-19 with acuted lamellae on inner margins.

Spiniform process on segment 10 very short, reaching only distal third of its segment; that on segment 11 short, reaching proximal third of the next segment. Convergent spiniform processes on segments 13 and 14. Base of stout spiniform process on segment 13 almost as wide as the length of its segment. Segment 20 is 3.6 times as long as wide, bearing a hook-like projection (less than the half length of penultimate segment) with a smooth hyaline membrane.

Antennule, antenna, mandible, maxillule, maxilla, maxilliped, and P1-P4 as described for female.

Right wing of fifth pediger with 1 tiny dorsal spinule and 1 ventral spine (Fig. 4B); left wing bearing 1 small spine (Fig. 4C).

Urosome: Urosomites nude dorsally and ventrally. First urosomite with thin spine on right side and fold on left side (Fig. 4E). Fourth urosomite slightly projected on right side. Right caudal ramus with wedge-like structure at disto-inner corner of ventral surface; medial margins of rami pilose (Figs 4E, 5A, B).

P5: Coxal segments with strong spines on caudal view; left and right basis with a lateral seta (Fig. 5D).

Left basis with a triangular protuberance on distal margin of frontal surface (Fig. 5C). Both left Exp1 and Exp2 pilose on medial margins (Fig. 5F), left Exp2 triangular, with its tip adorned with a small inner seta and spinules (Fig. 5F). Left Enp 1-segmented, distally feathered and as long as left Exp1 (Fig. 5C).

Right basis basally and distally projected: basal projection rounded whereas distal projection triangle-shaped; one semi-triangular sclerotization on caudal surface of right basis (Fig. 5D, E). Right Exp1 quadrangular in frontal view (Fig. 5C), one triangular fold and one rounded projection on caudal view (Fig. 5D). Right Exp2 1.6–1.8 times longer than wide and 1.9–2.0 times longer than aculeus, smooth in both frontal and caudal views (Fig. 5C, D). Aculeus inserted at distal third of the segment, pointed, unarmed, short (no longer than the width of the right Exp2). Terminal claw twice the length of Exp2 smoothly bent and ornamented with tiny spinules on inner margin (Fig. 5D). Right Enp one-segmented slightly longer than right Exp1 (Fig. 5C).

##### Molecular features.

The nucleotide sequence (607 bp) obtained for specimen MAGA-0156 (one adult male), identified as *Mastigodiaptomus cuneatus* sp. n. is shown below:

GGAGCCTGGTCAGGCATAGTAGGAACAGGCCTTAGAATGATTATTCGGATGGAGTTAGGACAAGCCGGGTCTTTAATTGGAGATGACCAAATTTATAATGTAGTAGTTACTGCTCATGCTTTTGTTATAATTTTTTTTATGGTGATACCTATTTTAATTGGGGGGTTTGGTAATTGGCTTGTTCCGTTAATATTAGGTGCAGCGGATATAGCTTTCCCTCGAATAAATAATATAAGATTTTGATTTTTATTGCCAGCTTTAGTCATATTGTTATCTAGGTCGCTTGTTGAAAGAGGGGCGGGAACAGGGTGAACTGTGTATCCCCCCCTGTCTAGCAACATTGCCCATGCTGGCAGGTCCGTTGATTTTGCTATTTTTTCGCTTCATTTAGCTGGGGTTAGGTCTATTTTGGGCGCAGTAAATTTTATTAGCACATTAGGAAATTTGCGGGCGTTTGGAATAATTTTAGATCGAATACCACTTTTTGCTTGAGCCGTTTTAATCACGGCTATCTTGTTATTGCTTTCTCTTCCTGTTTTAGCCGGGGCGATTACAATGCTTCTTACAGATCGGAACCTCAACTCAAGATTTTATGAT.

**Figure 6.**
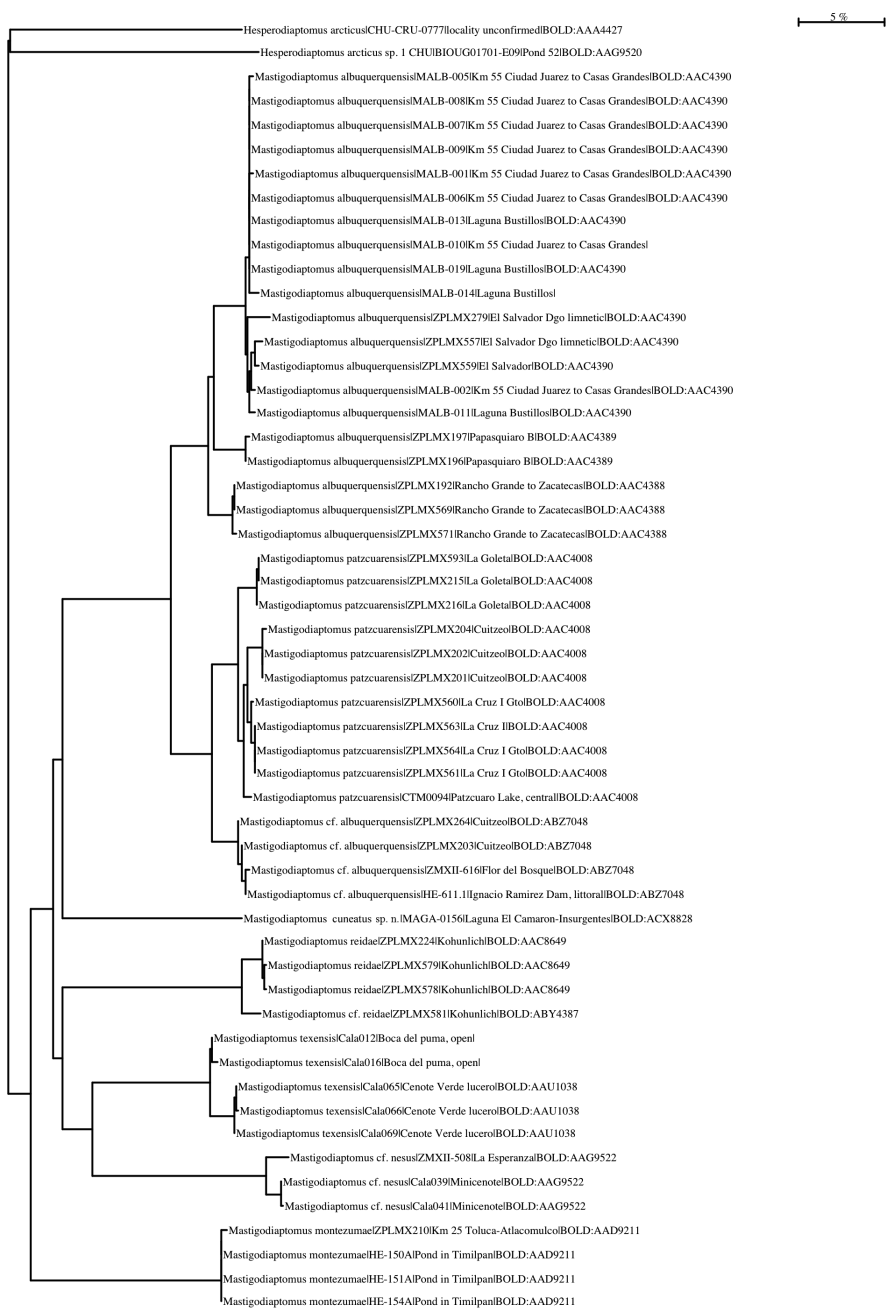
Maximum likelihood tree based on Kimura 2 parameter. The outgroup is *Hesperodiaptomus arcticus*. Numbers represent the specimen ID in BOLD or the GenBank Accession numbers.

The K2P maximum distance between the surveyed species reaching 5.52% (Table 3). The nearest neighbour of *Mastigodiaptomus cuneatus* sp. n. is *Mastigodiaptomus albuquerquensis* with 18.64% of genetic distance (Fig. 6).

**Table 3.** Sequence divergence (K2P) in the genus and species analysed.

	n	Taxa	Comparisons	Min Dist (%)	Mean Dist (%)	Max Dist (%)	SE Dist (%)
Within species	50	7	273	0	1.94	5.52	0.01
Within genus	54	2	1054	2.34	18.28	28.19	0.01

## Discussion

*Mastigodiaptomus cuneatus* sp. n. is assigned to the genus *Mastigodiaptomus* because it fulfils all the morphological generic criteria as given in Dussart and Defaye (1995), especially the left and right antennules of females and males and the ornamentation of coxa, basis and the endopodal or exopodal lengths of the fifth legs.

*Mastigodiaptomus cuneatus* sp. n. (from northern Mexico) appeared to be morphologically close to *Mastigodiaptomus amatitlanensis* (from Lago Amatitlán, Guatemala). Similarities between these species in females include the presence of one protrusion on the second urosomite and the bulbose lateral margins of genital double-somite. The similarities in males are the short aculeus and the lack of hyaline membrane on the right Exp2 of the fifth leg.

However, *Mastigodiaptomus cuneatus* sp. n. can be separated from *Mastigodiaptomus amatitlanensis* by the following features: the dorsal projection on the last pediger absent vs. present; the genital double-somite with vs. without a protrusion on the distal right side; on the genital double-somite, the right spine located at a higher level than the left spine vs. both left and right spines placed at same level; and the endopod of fifth leg long and 2-segmented vs. short and 1-segmented.

The males of these species show more morphological differences in the fifth leg: the caudal surface of the right basis is bulbose with the oblique, medial, angulose and curved cuticular process in *Mastigodiaptomus cuneatus* sp. n. in comparison to the rectangular basis with a transversal, distal, cuneiform lamella in *Mastigodiaptomus amatitlanensis*. In addition, the distal margins of the left and right endopods bear short setules in *Mastigodiaptomus cuneatus* sp. n. whereas in *Mastigodiaptomus amatitlanensis* these distal margins bear one slender seta. The aculeus on the right Exp2 is clearly straight in *Mastigodiaptomus cuneatus* sp. n. but short, distal and curved in *Mastigodiaptomus amatitlanensis*. The Exp2 is smooth in *Mastigodiaptomus cuneatus* sp. n. but in *Mastigodiaptomus amatitlanensis* an oblique ridge on the caudal surface is present. Finally, we assume that the wedge on the right caudal ramus present in *Mastigodiaptomus cuneatus* sp. n. is absent in *Mastigodiaptomus amatitlanensis* because there is no mention of a similar feature in Wilson (1941) or Wilson and Yeatman (1959).

Results related with the COI gene suggested that *Mastigodiaptomus cuneatus* sp. n. is genetically closest to *Mastigodiaptomus albuquerquensis* s. str. and the species recorded in Mexico with one sclerotization (similar to the half wing of a butterfly) on the right basis of fifth leg of males such as *Mastigodiaptomus patzcuarensis*. As previously discussed (see Gutiérrez-Aguirre et al. 2014), this particular sclerotization should not be used as a specific character because actually, it is shared by at least three different species. Therefore, in addition to the genetic distance and the sequences of the COI gene, some morphological features are suggested to separate them.

Whereas the distal margins of prosomites are pilose in females and males of *Mastigodiaptomus patzcuarensis*, these structures are nude in *Mastigodiaptomus cuneatus* sp. n and they have tiny spinules on lateral margins in *Mastigodiaptomus albuquerquensis*. The right Exp2 bearing one curved hyaline membrane and the aculeus is 2-3 times longer than the segment’s width in *Mastigodiaptomus albuquerquensis* and *Mastigodiaptomus patzcuarensis*; this Exp2 is nude with the aculeus shorter or as long as the segment’s width in *Mastigodiaptomus cuneatus* sp. n. There are no protrusions on the urosomites of males or females of *Mastigodiaptomus albuquerquensis* or *Mastigodiaptomus patzcuarensis*, but such structures do occur in *Mastigodiaptomus cuneatus* sp. n.

Sequences of the COI gene of *Mastigodiaptomus maya*, *Mastigodiaptomus suarezmoralesi*, *Mastigodiaptomus amatitlanensis*, *Mastigodiaptomus purpureus*, and *Mastigodiaptomus nesus* from their type localities or areas of their primary distribution, are not yet available for comparison and then the genetic distances between the species showed in Fig. 6 may change when the genetic sequences of the previous species can be added. However, until now, the lowest genetic distance in nearest neighbours of *Mastigodiaptomus* is between *Mastigodiaptomus patzcuarensis* and Mastigodiaptomus cf. albuquerquensis (3.36%), which probably are cryptic species (see Gutiérrez-Aguirre et al. 2014).

*Mastigodiaptomus* is considered a Neotropical genus and the species with the widest distribution are *Mastigodiaptomus albuquerquensis* (South of USA and North of Mexico), *Mastigodiaptomus patzcuarensis*, *Mastigodiaptomus montezumae* (Central Mexico), *Mastigodiaptomus nesus* (Caribbean and south eastern Mexico), and *Mastigodiaptomus texensis* (Texas and south eastern Mexico), whereas the species that are assumed to have restricted distribution or endemics are *Mastigodiaptomus reidae*, *Mastigodiaptomus maya*, *Mastigodiaptomus purpureus*, *Mastigodiaptomus amatitlanensis*, *Mastigodiaptomus suarezmoralesi* (see Gutiérrez-Aguirre and Cervantes-Martínez 2013) and, probably, *Mastigodiaptomus cuneatus* sp. n.

## Conclusion

Morphological and genetic differences were found when *Mastigodiaptomus cuneatus* sp. n. was compared with the ten known *Mastigodiaptomus* species, particularly in the female and male urosomes, the male right antennule and fifth legs, and in the COI gene sequence. This report increases the number of recognized species of *Mastigodiaptomus* to eleven. *Mastigodiaptomus cuneatus* sp. n. appears to be part of the *Mastigodiaptomus albuquerquensis* complex.

### Key to species of *Mastigodiaptomus*

**Males**

**Table:** 

1	Spiniform process on segment 16 of right antennules strongly developed almost as long as its segment width; right basis of P5 with one basal subrectangular protuberance	***Mastigodiaptomus reidae***
–	Spiniform process on segment 16 of right antennules reduced or absent; right basis of P5 with basal rounded protuberance or without basal protuberance	**2**
2	Right basis of P5 with only one lobular protuberance on basal-medial margin, no chitinous lamella or lamellae (on caudal view)	**3**
–	Right basis of P5 with chitinous lamella or lamellae and with or without rounded protuberance on basal-medial margin (on caudal view)	**4**
3	Lobular protuberance of right basis of P5 large; right Exp2 of P5 with two semicircular transverse lamellae, and one longitudinal “Y” shaped ridge; antepenultimate antennular segment with a fang-like process	***Mastigodiaptomus montezumae***
–	Lobular protuberance of right basis of P5 short; right Exp2 of P5 with a low rounded protuberance on outer margin; antepenultimate antennular segment with wide knob-like process	***Mastigodiaptomus maya***
4	Right basis of P5 with one chitinous lamella	**5**
–	Right basis of P5 with more than one chitinous lamella	**8**
5	Chitinous lamella of right basis of P5 semi-circular, on medial margin	**6**
–	Chitinous lamella of right basis of P5 cuneiform, transversal; or semi-triangular, on caudal side	**7**
6	Right Exp2 of P5 with two short semi-circular lamellae (one medial and one lateral); aculeus with 50% of the length of its segment; short spiniform process on antennular segment 14	***Mastigodiaptomus purpureus***
–	Right Exp2 of P5 with one long quadrangular lamella (on caudal side) aculeus with 70–90% of the length of its segment; long spiniform process on antennular segment 14	***Mastigodiaptomus nesus***
7	Caudal surface of right basis of P5 with one triangular protuberance on distal margin, and one rounded protuberance on basal-medial margin; right Exp2 of P5 nude; right furcal ramus with one wedge-like structure at disto-inner corner of ventral surface	***Mastigodiaptomus cuneatus* sp. n.**
–	Caudal surface of right basis of P5 without protuberances, almost rectangular; right Exp2 of P5 with one straight ridge, obliquely directed	***Mastigodiaptomus amatitlanensis***
8	Left Exp2 of P5 distally truncated and denticulate; aculeus inserted subterminally on Exp2 of P5	***Mastigodiaptomus texensis***
–	Left Exp2 of P5 distally attenuated (triangular-shape) aculeus inserted on the second third of Exp 2 of P5	**9**
9	Aculeus shorter than the length of right Exp2 of P5 with long spinules on medial margin; two short semi-circular lamellae on medial margin of right Exp2 of P5; second to forth urosomites with denticles on dorsal surfaces	***Mastigodiaptomus suarezmoralesi***
–	Aculeus subequal or longer than the length of right Exp2 of P5 with short spinules on medial margin; one long curved hyaline lamella on right Exp2 of P5; dorsal surfaces of urosomites nude	**10**
10	Short spines on rostrum: 2.2–3.0 times longer than width; cuticular surfaces of prosomites nude; 1.37–1.82 mm of body length including furcal ramus	***Mastigodiaptomus albuquerquensis***
–	Long spines on rostrum: 3.5–5.0 times longer than width; hair-like setae on ventral and distal margins from second to fifth prosomites; 0.92–1.1 mm of body length including furcal ramus	***Mastigodiaptomus patzcuarensis***

**Females**

**Table:** 

1	Hair-like setae only on medial margin of furcal ramus	**2**
–	Hair-like setae on both, medial and lateral margins of furcal ramus	**3**
2	Symmetric genital double-somite, almost parallel lateral margins with short spines on left and right margins	***Mastigodiaptomus purpureus***
–	Asymmetric genital double-somite, bulbose lateral margins with short spines on left and right margins	***Mastigodiaptomus reidae***
3	Symmetric genital double-somite, almost parallel lateral margins; two-segmented Enp of P5 bearing one apical spinule longer than the endopodal width	**4**
–	Asymmetric genital double-somite, bulbose lateral margins; one or two-segmented Enp of P5 bearing two apical spinules shorter than the endopodal width	**5**
4	Enp of P5 longer than the inner margin of Exp1 of P5; long lateral sensilla of coxal segment of P5	***Mastigodiaptomus texensis***
–	Enp of P5 shorter than the inner margin of Exp1 of P5; short lateral sensilla of coxal segment of P5	***Mastigodiaptomus maya***
5	One or two urosomites with a chitinous protuberance on the right disto-lateral corner	**6**
–	Urosomites with straight posterior margins, no protuberances are present	**7**
6	Second urosomite with a spine-like protuberance on the right disto-lateral corner; one-segmented Enp of P5	***Mastigodiaptomus amatitlanensis***
–	Genial double-somite and second urosomite with chitinous protuberance on the right disto-lateral corner; two-segmented Enp of P5	***Mastigodiaptomus cuneatus* sp. n.**
7	Genital double-somite produced, or curved and wrinkled on the right disto-lateral margin	**8**
–	Genital double-somite straight on the right disto-lateral margin	**9**
8	Two-segmented Enp of P5, as long as medial margin of Exp1; genital double-somite curved and wrinkled on the right disto-lateral margin	***Mastigodiaptomus suarezmoralesi***
–	Two-segmented Enp of P5, shorter than the half length of medial margin of Exp1; genital double-somite produced on the right disto-lateral margin	***Mastigodiaptomus montezumae***
9	One-segmented Enp of P5; left and right spines of genital double-somite inserted at same level; ventral spine of right wing (of last prosomite) directed to the posterior region of the body	***Mastigodiaptomus nesus***
–	Two-segmented Enp of P5; right spine of genital double-somite inserted more anteriorly than left spine; ventral spine of right wing (of last prosomite) directed to the dorsal region of the body	**10**
10	Short spines on rostrum: 1.5–3.6 times longer than width; tiny spines on ventral surface of each prosomites; 1.47–1.87 mm of body length including furcal ramus	***Mastigodiaptomus albuquerquensis***
–	Long spines on rostrum: 3.6–4.0 times longer than width; hair-like setae on ventral and distal margins from second to fifth prosomites; 0.9–1.3 mm of body length including furcal ramus	***Mastigodiaptomus patzcuarensis***

## Supplementary Material

XML Treatment for Mastigodiaptomus cuneatus

## References

[B1] Alekseev V Dumont HJ Pensaert J Baribwegure D Vanfleteren JR (2006) A redescription of *Eucyclops serrulatus* (Fischer, 1851) (Crustacea: Copepoda: Cyclopoida) and some related taxa with a phylogeny of the *E. serrulatus*-group. Zoologica Scripta 35(2): 123–147. doi: 10.1111/j.1463-6409.2006.00223.x

[B2] Bowman TE (1986) Freshwater calanoid copepods from the West Indies. Syllogeus 58: 237–246.

[B3] Dussart D Defaye D (1995) Copepoda, Introduction to the Copepoda. SPB Academic Publishing, Belgium, 277 pp. doi: 10.1002/iroh.19960810306

[B4] Folmer O Black M Hoeh W Lutz R Vrijenhoek R (1994) DNA primers for amplification of mitochondrial cytochrome *c* oxidase subunit I from diverse metazoan invertebrates. Molecular Marine Biology and Biotechnology 3: 294–299. 7881515

[B5] Gutiérrez-Aguirre MA Cervantes-Martínez A (2013) Diversity of freshwater copepods (Maxillopoda: Copepoda: Calanoida, Cyclopoida) from Chiapas, Mexico with a description of *Mastigodiaptomus suarezmoralesi* sp. nov. Journal of Natural History 47(5–12): 479–498. doi: 10.1080/00222933.2012.742587

[B6] Gutiérrez-Aguirre MA Cervantes-Martínez A Elías-Gutiérrez M (2014) An example of how barcodes can clarify cryptic species: the case of the calanoid copepod *Mastigodiaptomus albuquerquensis* (Herrick). PLoS ONE 9(1): e85019. doi: 10.1371/journal.pone.0085019 24465470 10.1371/journal.pone.0085019 PMC3897401

[B7] Hołyńska M (2000) Revision of the Australasian species of the genus *Mesocyclops* Sars, 1914 (Copepoda: Cyclopidae). Annales Zoologici 50(3): 363–447.

[B8] Huys R Boxshall GA (1991) Copepod Evolution. The Ray Society, London, 468 pp.

[B9] Kimura M (1980) A simple method of estimating evolutionary rate of base substitutions through comparative studies. Journal of Molecular Evolution 16(2): 111–120. doi: 10.1007/BF01731581 7463489 10.1007/BF01731581

[B10] Montero-Pau J Gomez A Muñoz J (2008) Application of an inexpensive and high-throughput genomic DNA extraction method for the molecular ecology of zooplanktonic diapausing eggs. Limnology and Oceanography-Methods 6: 218–222. doi: 10.4319/lom.2008.6.218

[B11] Montiel-Martínez A Ciros-Pérez J Ortega-Mayagoitia E Elías-Gutiérrez M (2008) Morphological, ecological, reproductive and molecular evidence for *Leptodiaptomus garciai* (Osorio-Tafall 1942) as a valid endemic species. Journal of Plankton Research 30(10): 1079–1093. doi: 10.1093/plankt/fbn067

[B12] Prosser S Martínez-Arce A Elías-Gutiérrez M (2013) A new set of primers and some methodological improvements for COI amplification in freshwater microcrustaceans. Molecular Ecology Resources 13(6): 1151–1155. doi: 10.1111/1755-0998.12132 23795700 10.1111/1755-0998.12132

[B13] Quiroz-Vázquez P Elías-Gutiérrez M (2009) A new species of the freshwater Cladoceran genus *Scapholeberis* Schoedler, 1858 (Cladocera: Anomopoda) from the Semidesert Northern Mexico, Highlighted by DNA Barcoding. Zootaxa 2236: 50–64. doi: 10.15468/mn7p62

[B14] Van de Velde I (1984) Revision of the African species of the genus *Mesocyclops* Sars, 1914 (Copepoda: Cyclopidae). Hydrobiologia 109(1): 3–66. doi: 10.1007/BF00006297

[B15] Wilson MS (1941) New species and distribution records of diaptomid copepods from the Marsh collection in the United States National Museum. Journal of the Washington Academy of Sciences 31: 509–515.

[B16] Wilson MS Yeatman HC (1959) Free-living Copepoda. In: Edmonson WT (Ed.) Ward’s and Whipple’s Freshwater Biology. John Wiley and Sons, New York, 735–861.

